# “Family planning in Rwanda is not seen as population control, but rather as a way to empower the people”: examining Rwanda’s success in family planning from the perspective of public and private stakeholders

**DOI:** 10.1186/s40834-018-0072-y

**Published:** 2018-11-20

**Authors:** Hilary M. Schwandt, Seth Feinberg, Akrofi Akotiah, Tong Yuan Douville, Elliot V. Gardner, Claudette Imbabazi, Erin McQuin, Maha Mohamed, Alexis Rugoyera, Diuedonné Musemakweli, Cliff Wes Nichols, Nelly Uwajeneza Nyangezi, Joshua Serrano Arizmendi, Doopashika Welikala, Benjamin Yamuragiye, Liliana Zigo

**Affiliations:** 1 Fairhaven College, Western Washington University, 516 High Street MS 9118, Bellingham, WA 98225 USA; 2 Western Washington University, 516 High Street, Bellingham, WA 98225 USA; 3 Wheaton College Massachusetts, 26 E. Main Street, Norton, MA 02766 USA; 4 Southern Methodist University, PO Box 750100, Dallas, TX 75275 USA; 5 New College of Florida, 5800 Bay Shore Rd, Sarasota, FL 34243 USA; 6 INES, P.O.Box: 155, Ruhengeri, Rwanda; 7 Truman State University, 100 E Normal St, Kirksville, MO 63501 USA; 8 Austin College, 900 N. Grand Ave, Sherman, TX 75090 USA; 9 University of Maryland Baltimore County, 1000 Hilltop Cir, Baltimore, MD 21250 USA; 10 American University, 4400 Massachusetts Ave NW, Washington, DC 20016 USA

**Keywords:** Rwanda, Family planning, Family planning program

## Abstract

**Background:**

Rwanda has made significant strides in improving the health of its people, including increasing access to and use of family planning. Contraceptive use has increased from 17% to 53% in just one decade, from 2005 to 2015.

**Methods:**

The data consist of 13 in-depth interviews conducted with family planning program experts in Rwanda to better understand the mechanisms for success, elucidate remaining challenges, speculate on the future of the program, and discuss potential applicability for translating aspects of the program in other settings.

**Results:**

All respondents first noted the positive aspects of government will, leadership, and management of the family planning program when asked to describe the reasons for success. The challenges that loomed the largest for the program were service accessibility for rural Rwandans, adolescent access to and use of contraceptives, opposition from religious institutions, as well as inadequate human resources and funding. These challenges were openly acknowledged and are in the process of being addressed.

**Conclusion:**

The importance of government leadership and focus in the success of Rwanda’s family planning program was prominent. All positive aspects of the program are based upon the strong foundation the government has built and nurtured. Since innovation is welcomed and program evaluation is considered essential, the outlook for Rwanda’s family planning program is favorable. The issues that remain are common and persistent challenges for family planning programs. Other nations could learn tangible practices from Rwanda’s success and follow Rwanda’s efforts to mitigate the remaining challenges.

## Plain English summary

Rwanda has made significant strides in improving the health of its people, including increasing access to and use of family planning. Contraceptive use increased from 17% to 53% in just ten years, from 2005 to 2015. This study aimed to better understand the factors that made this increase in contraceptive use in Rwanda possible, the challenges that remain, and the possibility of translating aspects of the success in other settings. The data for this study come from 13 interviews with experts in the family planning program in Rwanda. All of the study participants first discussed the important role of the government when asked to describe the reasons for success. All positive aspects of the program are based upon the strong foundation the government has built and nurtured. The challenges that loomed the largest for the program were access to services for rural Rwandans, adolescent access to and use of contraceptives, opposition from religious institutions, as well as inadequate human resources and funding. All of the challenges were openly acknowledged and are in the process of being addressed. Since innovation is welcomed and program evaluation is considered essential, the outlook for Rwanda’s family planning program is favorable. Other nations could learn tangible practices from Rwanda’s success and follow Rwanda’s efforts to mitigate the remaining challenges.

## Introduction

Individual families and broader communities are empowered when women have the ability to control the frequency and timing of births. Family planning is therefore a central component of a healthy and sustainable society. Both for specific measures like infant or maternal mortality, to broader indicators of employment, education and life-expectancy, a highly-functioning family planning program is beneficial to the overall health of a nation [1].

The government of Rwanda has embraced family planning as a central component of development [2]. Beyond the timing and limiting of children, the government views family planning as a vehicle to better health through decreased maternal, infant, and child mortality [3].

Rwanda has made measurable success in bringing family planning information and accessibility to its citizens. Targeted governmental policy implementation has thus far yielded impressive gains over the last 10 years in modern contraceptive use. Following a set of top-down policy mandates from the federal government, contraceptive use in Rwanda has increased by 17% to 53% over a 10-year period [4]. This growth is remarkable and an obvious outlier in family planning programs globally, particularly in sub-Saharan Africa which has typically lagged behind more general global increases in family planning accessibility [5].

It is important to note, however, that the gains in contraceptive use still reflect only 53% of women utilizing contraceptive methods [4]. This highlights a critical need to continue capacity building efforts. The Rwandan Government maintains an aggressive approach to the stated goal of reaching middle-income country status, and views family planning as an integral part of the strategy to grow the nation’s economy. Understanding potential barriers and limitations that remain in this family planning program has obvious benefit to individual women, families, communities, the nation, and beyond. Externally, Rwanda’s increased family planning capacity trends may provide potential models for other countries, both in the sub-Saharan African region and globally as well [6].

Given the importance of family planning in improving the health of children, mothers, fathers, families, and nations (Cleland et al., 2012) – it is important to not only measure the success but also to understand the mechanisms generating this success. The research goal is to use qualitative interviews to learn from the current generation of high-level government and private sector family planning experts working to implement and assess the nation’s family planning program to shed light on the following: key factors promoting this measured progress, potential determinants that are limiting the continued success of Rwanda’s family planning program, the future outlook for the program, as well as the possibility of translating some of the success in other national contexts.

## Methodology

To address this research topic, data came from 13 in-depth interviews to obtain information from experts, academics, government employees, and those from the private sector, working in the family planning program in Rwanda. The topic guide included questions about the study participant’s background, family planning program strengths and weaknesses, anticipated future directions, as well as the possibility of translating aspects of the family planning program in different contexts. Two of the authors (including one native Kinyarwanda speaker) conducted each interview in English at the offices of the key informants or other locations identified as conducive by the interviewees.

The interviews averaged 45 min in duration with a range of 20 to 90 min. Interviews were audio recorded when permission was received from participants. This was the case for twelve of the interviews. The recordings were then transcribed verbatim. Data analysis was guided by the thematic content analysis approach and executed using Atlas.ti software [7] and group level matrices [8]. Institutional Review Boards at Western Washington University in Bellingham, Washington and the Ministry of Education in Kigali, Rwanda approved the study in advance of data collection.

## Results

### Successes

When asked to describe the motivating factor behind Rwanda’s successful family planning program, all study participants first mentioned the strong impact of the government. In fact, all positive aspects mentioned as contributing to the success of the program could be traced back to the government’s political will, strong leadership, innovation, funding, and evaluation. Importantly, respondents described collaboration across sectors driven with clear intent from the highest levels of government.

### Political will

When describing the role of the government in making the family planning program in Rwanda such a success, the study participants noted that the government has a “clearly defined agenda” and makes family planning “a national priority” to “reduce maternal mortality and...decrease poverty.” Participants also noted how there is a positive empowering message to encourage thoughtful childbearing, rather than simply restricting people from procreation: “the message that has been passed on is not about not having children, but it’s about having the right number of children you can look after.” In other words, “Rather than having more children, improve on the ones you have.” This can serve to motivate citizens by placing responsibility for family planning outcomes within their locus of control. A common response from interviews highlighted that the government is not there to curb population growth, but rather to give people the tools necessary to develop as individuals and families, and therefore, advance the entire nation: “family planning in Rwanda is not seen as population control, but rather as a way to empower the people.”

### Strong leadership and innovation

Many respondents noted the strong and dedicated government leadership as also being key to the program’s success. Interviewees emphasized the leadership of President Paul Kagame: “Everything starts at the higher level…his Excellency, our president of this country. He is really committed to family planning.” The President’s commitment is felt through his public support of family planning, policies implementation, and involvement in the sustained growth of the program.

The government receives most of the recognition for innovative efforts in Rwanda, suggesting that innovation and strong leadership go hand in hand. “One of the successes in Rwanda is...the political people are engaged in the system. The political weight is a success because they will accept innovation, they will accept whatever we want to implement, they will be flexible.” Consensus from respondents suggests that this results-oriented approach promoted from the government to obtain quality outcomes manifests in both planning and practice.

### Funding and evaluation

The Rwandan Government has included the family planning program in the national budget and partially funds the program. The government’s role in funding has increased over the last decade. Partially due to financial buy-in, the Rwandan government strongly believes research is necessary to inform evidence-based program improvements. The Rwandan government monitors the family planning programs at all levels, as noted by one participant: “Performance indicators go all the way up to the President of Rwanda. There is a lot of accountability.” There is accountability as well as competition: “If a district is doing well they are rewarded and lower performing district officials come to visit the successful districts to learn from them and apply the lessons they learned to the programs in their own districts. The competition between districts helps them to stay motivated.” As a result, districts are motivated to perform well and have the support from the government to do so.

### Collaboration

Respondents stressed that intentional and inclusive collaboration, initiated by the government within the government and with all stakeholders, has been essential in meeting the goals of the program. One way that the government has achieved this goal is through the creation of a Family Planning and Technical Working Group (FPTWG) that includes partners from every level within the government and the private sector. Once a month, all stakeholders come together to discuss successes, challenges, future priorities, and solutions. “...[The] technical working group helps the government to do the coordination role, [and] the harmonization of what is being done by different partners…”. Additionally, the FPTWG has increased program efficiency by minimizing duplication of efforts and utilizing the strengths of all key players.

### Government ownership

While the study participants recognized the power of collaboration with all partners, public and private, respondents stressed the importance of government ownership and guidance of the entire program for consistency and sustainability: “even public institutions at the level of the village can provide methods without the presence of some midwife…or doctor working with NGOs who will come to the village once a week or don’t. The program is under the government for management. This is the key success of Rwanda.”

### Challenges

Study participants openly acknowledged challenges faced by Rwanda’s family planning program, including the following issues: reaching rural Rwandans with services, adolescent access to and use of services, funding, human resources, and opposition from religious institutions. Each challenge identified has been addressed, at least partially, with innovative solutions.

### Rural residence

“We have difficult physical terrain in Rwanda, and some people have to travel long distances to seek out services...”. Due to the agrarian life of most Rwandans, the distance Rwandans have to travel for family planning services was noted as a challenge by many participants. In addition, residence also affects what methods are available to individuals. Permanent methods can be more difficult to obtain in rural areas because these services are only offered at the district hospitals or referral hospitals in the urban centers. To increase access to services for rural Rwandans the government decentralized and added more health centers. The government also started a community health worker program to increase the reach of family planning services to those in every area of the country.

### Decentralization and infrastructure

Decentralization transfers power from the central government to local authorities. In terms of family planning, decentralization has increased access to services for rural populations by bringing the infrastructure closer to the people: “…The plan is to have a health post at each cell, if …each cell has a health post…has a health provider trained to provide family planning, there will be no problem with…accessibility.” Decentralization has also enabled communities to identify their needs and, therefore, tailor services accordingly. “Officials are directly involved in decision making and have the ability and capacity to evaluate programming, identify gaps, and work to fill those gaps.”

### Community health workers

With their emic perspective, community health workers (CHWs) act as liaisons between higher levels of government and communities. CHWs distribute short-acting methods such as condoms, pills, injectables, and cycle beads. One respondent explained the impact of equipping CHWs with the contraceptives themselves: “…They (CHWs) are neighbors of these people. [If they can provide half of the methods], this problem of geographical accessibility is completely destroyed.” As elected volunteers, CHWs understand and are respected by the communities they serve. Respondents praised the CHW program: “…Community health workers…and health workers in health facilities, those are the champions [of the] family planning program. They are the people leading, supporting, helping the community.”

### Adolescent family planning use

Rwandan culture largely indicates that youth abstain from sexual intercourse until marriage. This belief results in stigma towards unmarried youth accessing contraceptive services. The stigma that exists may preempt unmarried adolescents from pursuing methods to begin with, make them hesitate to request methods while at a clinic, or have their access to methods denied at the provider level. Even among some at the central level there was the perception that providing family planning to unmarried youth is inappropriate and encourages sex. “At their age they don’t need family planning...what they need is awareness.”

The belief that traditional social expectations for adolescents conflict with government’s promotion of family planning among youth was discussed by several participants. “When we give [FP methods] to adolescents then we are encouraging them to practice sex...but when you look on the real culture of the country, sex is not...accepted.” Most respondents believed that expansion of family planning services to unmarried adolescents and youth is necessary since they make up a significant share of the population and are currently underserved – and were hopeful that this area of need will be met in the future. “It’s not really fully supported...So, it is of course a challenge…But maybe one day…it will be on a level where, we will say now adolescents can get accessible (family planning) products as well.”

Another emerging theme was the challenge of finding the physical space to provide adolescents with services and information. Traditional avenues of receiving care offer little privacy to unmarried adolescents who are heavily influenced by the stigma around family planning. Officials are aware of this gap in service delivery, and have several ongoing initiatives in place. Stand-alone “youth centers,” and “youth corners” integrated into existing health facilities are projects that aim to give adolescents a safe space to receive sexual health information and family planning methods. While many districts have facilities like these, not all are currently operational, and the initiative is still growing as part of Rwanda’s overall strategy to reach young people.

### Funding

The Rwandan Government partially funds the family planning program and the government’s role in funding has increased over the last decade; however, the majority of funding still comes from international partners. Study participants noted how the Rwandan government will need to continue finding partners to help provide funds, as well as increasing its internal budgetary commitment to sustain the success of the family planning program. “Most of the funding to family services has been…from external donors… and that is, ... a major challenge. Although the government has been increasing its budget support to the health sector, it will take considerable time until they at least break even to what our funders are providing.”

### Religious institutions

Many respondents noted religious opposition as a barrier to success as 40% of the health facilities in Rwanda are run by the Catholic Church. In Catholic health facilities, only traditional contraceptive methods are offered. Modern methods are not provided. In response to the void created in these Catholic-run health facilities, the Rwandan Government has created secondary health posts located adjacent to or near the faith-based health facilities. Respondents commented that there is the belief that regardless of religion, all people should be educated about all types of contraception and have access to such methods so they can make informed choices.

### Human resources

Study participants noted human resources as both a strength and a challenge for Rwanda’s family planning program. They expressed the need to increase the number of doctors, nurses, and community health workers, as well as the capacity of all health personnel through extensive, yet efficient, training. Participants also reported high turnover rates from health workers and the negative effect this has on the success of the program, “...another challenge is related to high turnover of service providers...currently there are some services...no one can provide, for instance, permanent methods because the one who used to provide that, he shifted to another hospital or another clinic.” Despite these challenges, participants also consistently highlighted the increase in training on family planning across all health provider types – so any health provider can and will inform patients about family planning regardless for the reason for their visit to the health center.

### Family planning future in Rwanda

When asked about the future of the family planning program in Rwanda, one participant responded: “I wish that the CPR would increase, I wish the TFR would decrease, I wish 0 unmet need for family planning. This is my dream.” Most participants noted the need to continue to expand on the successes, acknowledge and address the challenges, and to maintain a working attitude. As another participant notes: “I call it [family planning] a journey. It is not something you achieve and finish and go. Because human life continues. People still continue.”

### Translation

Participants in the study agreed that aspects of Rwanda’s successful family planning program can be translated to other countries and that “there is no magic”: “Well, I think it is tricky to copy and paste programs into other countries with different contexts. But there are specific innovations made in Rwanda that could be tailored to other countries’ contexts.” When participants were asked if the family planning program in Rwanda could be translated to other places, they were eager to explain the parts of the program that could help other countries succeed as well. As one participant explained: “we were not written the same way.” Thus, it is difficult to implement all parts of the Rwandan family planning program in other countries, but other countries can learn from specific parts of the program.

One of the key aspects discussed by all participants was government leadership and the integration of family planning into multiple aspects of society. Another element that participants’ thought could be translated into other countries’ family planning programs was the utilization of community health workers. As one participant stated enthusiastically: “We have the community health workers in Rwanda, who have done an incredibly amazing job. They are the ones who…increase the highest percentage of users of family planning...That’s something other countries can learn from us.”

## Discussion

Family planning is instrumental in development, as has been the case for Rwanda. This study sought to better elucidate the success of Rwanda’s family planning program, in light of global improvement in family planning programs, and lack of improvement in programs in the sub-Saharan Africa region [5], so other nations can learn from Rwanda’s success. Key informants at the central level shed light on the importance of government leadership and political will in being the main contribution to success for the family planning program in Rwanda [6, 9–11]. Coordination and integration between family planning stakeholders, which has been facilitated by political will [2, 6, 12], has greatly contributed to the success as well [10].

Participants also openly discussed the challenges of sustainability and long-term funding [10], continued efforts to increase accessibility in rural areas, addressing the adolescent access gap, and barriers from Catholic Church providers as current foci of the program. The key informants were very open to discussing the program challenges and were hopeful about the ongoing proactive efforts to address each of the identified challenges.

Experiences in other contexts, inside and outside sub-Saharan Africa, indicate that political will [9, 10, 13] and international support to integrate family planning into the development goals of the nation greatly contribute to its success [14–16]. Post-1994 Rwandan governance is unique in terms of the high percentage of female legislators, consistently ranking highest in the world [17]. There may be important lessons from enhanced diversity in the political leadership that directly impacts the focus of politics that the global health community can learn from.

Funding and support from the government is necessary, and is not solely the job of foreign investors. Doing so creates internal accountability and, therefore, effort to monitor and evaluate the impact of inputs on program outputs. A willingness to fund the program [16], to evaluate the program, and to use the results of the evaluation to improve the program are key factors for success. An openness to evaluation allows for openness to change through innovation. In this study in Rwanda, all of the challenges mentioned were being actively responded to through innovative ideas – some with more success than others, but all with ongoing plans for a proactive response.

Coordination between governments, within government sectors, with private actors, and NGOs is a key factor to the success of Rwanda’s family planning program [6, 10, 18]. As has been seen in other contexts, coordination of the family planning at all levels increases the success of the program as well as the accessibility of the services to clients [13, 16]. While usually successful, most countries have only tried integrating family planning with HIV services [19] or childhood immunization [20]. Rwanda’s integration of family planning with other services has been more pervasive [10].

A successful family planning program needs to address the challenge of geographic barriers to access, as Rwanda has begun to do with its decentralized program structure. Many study participants pointed to the key role the community health worker program has been to the success of contraceptive access in Rwanda. Experience has shown the net benefit of community health worker models of contraceptive provision [13, 16]. Research shows that community health workers, with training and under supervision, can provide all short and some long term methods [21]. It is possible that the way Rwanda selected community health workers, through community election, was part of the success in this arena, as this is uncommon [10].

This study, as well as others, identified stigma as a major barrier to youth’s use of family planning services and methods [18, 22, 23]. This is an issue in many contexts, even in areas with low fertility and well established family planning programs [24]. While Rwanda’s efforts to combat stigma are still in the early stages, other contexts seeking to address this difficult dilemma might consider provision of nontraditional spaces for youth to receive family planning counseling and methods, as has been also noted by providers in South Africa [25].

Religious opposition to family planning is a common barrier to family planning programs. Experience in Iran and Indonesia has shown that consultation with Muslim religious leaders, invitations to training and consulting, and requests to promote family planning to the populace was successful in combating religious opposition [13, 26]. More recent Muslim religious leader training in Jordan showed positive impacts of training on religious leader knowledge, resistance, and sharing of raised awareness with congregants [27]. In Rwanda, the government has engaged in meetings and discussions with religious leaders in the nation regarding the topic of family planning [10]. In addition, the government has made the innovative addition of health facilities nearby religious health facilities that are independent of the church to allow for the availability of modern contraceptives in places that would otherwise be lacking.

There are a few limitations of this research. As English is not the first language of any of the key informants, there was concern regarding communication barriers between the interviewers and the respondents. Additional limitations include the snowball sampling method. Key informants were chosen on behalf of who recommended them as well as their accessibility and availability. Strengths of this study included the participation of all researchers in the research process, double coding, and synthesis of the results. Another strength was the inclusion of Rwandan researchers on the research team.

The dearth of research on the successful family planning program in Rwanda is of concern. It is recommended that additional research be conducted on this program in order to also contribute to the literature on this critical topic. This study focused on interviewing national level stakeholders. It is recommended that future studies examine the successes of the family planning program with stakeholders at other levels, such as at the district or community level, the family planning service providers, and the beneficiaries of the program.

## Conclusion

Rwanda has made impressive gains in contraceptive use; however, contraceptive use is not the goal in Rwanda, but rather a means to “empower the people.” By acknowledging ongoing challenges, interviewees hoped to bring their nation closer to this goal. By sharing Rwanda’s successes, respondents hoped Rwanda’s innovations could be translated to other countries. Add strong political will and understanding a country’s context, and respondents were confident success would follow. Family planning is intertwined with other areas of health, development, poverty reduction, and empowerment in Rwanda. Thus, sharing Rwanda’s innovations, knowledge, and experiences with family planning, the country with the highest family planning program effort score [5], has the potential to create change beyond the nation’s borders. As one participant sagely said: “When one country in the world is effected, the rest of the countries are pulled down. So, when a country is pulled up, the others are lifted...”
